# Quantitative Analysis of the Instant and Persistent Inhibition Effects of Maternal Poliovirus Antibodies on the Immune Response in a Phase IV Trial of a Sabin Strain-Based Inactivated Poliovirus Vaccine

**DOI:** 10.3390/vaccines12020217

**Published:** 2024-02-19

**Authors:** Qiongzhou Yin, Yan Zheng, Zhifang Ying, Jingyu Li, Ya Jiang, Wenmei Bao, Youjian Dou, Yi Pu, Jin Lei, Haitao Yang, Ruiju Jiang, Yan Deng, Zhimei Zhao, Jing Pu, Jing Yang, Yadong Li, Min Xu, Wei Cai, Yanchun Che, Li Shi

**Affiliations:** 1 Institute of Medical Biology, Chinese Academy of Medical Science & Peking Union Medical College, Kunming 650118, China; yinqiongzhou@163.com (Q.Y.);; 2 Vaccine Clinical Research Center, Yunnan Center for Disease Control and Prevention, Kunming 650022, China; 3 Division of Respiratory Virus Vaccines, National Institutes for Food and Drug Control, Beijing 100050, China; 4 Mile Center for Disease Control and Prevention, Mile 652399, China; 5 Gejiu Center for Disease Control and Prevention, Gejiu 661000, China

**Keywords:** maternal antibody, inactivated poliovirus vaccine, Sabin strain, primary immunization, booster immunization, inhibition effects, quantitative analysis

## Abstract

Background: An inactivated poliomyelitis vaccine made from Sabin strains (sIPVs) has widely been used in China since 2015. However, the quantitative data on the instant and persistent inhibition effects of maternal poliovirus antibodies on the immune response to sIPV priming and booster vaccination have not been available yet. Objective: In this study, we aim to explore and quantify the instant and persistent inhibition effect of maternal poliovirus antibodies on the immune response elicited by sIPV primary and booster vaccination. Methods: The immunogenicity data consisting of the days 0 and 30 after the prime and booster vaccination of the sIPV in a phase IV trial were pooled for a quantitative analysis of the inhibition effect of maternal poliovirus antibody. The geometric mean ratio (GMR) was calculated using linear regression models, representing that every 2-fold higher maternal poliovirus antibody titer may result in a (1-GMR) lower postimmunization antibody titer. Results: The GMRs for poliovirus types 1, 2, and 3 were 0.79 (0.77–0.82), 0.85 (0.81–0.89), and 0.87 (0.83–0.91) at 30 days after the priming series, 0.86 (0.83–0.89), 0.81 (0.76–0.85), and 0.86 (0.80–0.93) at one year after the priming series, and 0.96 (0.94–0.99), 0.89 (0.86–0.93), and 0.98 (0.93–1.03) at 30 days after the booster dose. The inhibition effect continued to exist until the booster dose 1 year later, and such a persistent inhibition effect was almost attenuated for poliovirus types 1 and 3, and partly reduced for type 2 at 30 days after the booster dose. Conclusion: A wider interval between the four sIPV doses might be a consideration for reducing the effect of maternal antibodies and subsequently eliciting and maintaining higher antibody levels to protect against poliovirus transmission and infection at the final stage of polio eradication in the global world. This study’s clinical trial registry number is NCT04224519.

## 1. Introduction

To reach the final target of polio eradication worldwide, the inactivated poliomyelitis vaccine made from Sabin strains (sIPVs) plays an important role. Its advantage is in the biosafety requirements for production compared with the conventional IPV made from the wild-type poliovirus strains (cIPVs), which helps to reduce the total production costs and becomes a rational option in low- and middle-income countries [1]. However, several studies revealed that the immune response to IPV might be inhibited by maternally derived antibodies [2,3,4,5].

Maternal antibodies are antibodies transferred to infants through the placenta during the third trimester of pregnancy and provide passive immunity to the newborns from infections during the first few months of life [6]. However, passively acquired maternal antibodies have been proven to somewhat inhibit the immune response to infant vaccination with pertussis vaccines [7,8], hepatitis B vaccines [9], and cIPVs, which has widely been recognized to exhibit the greatest inhibition effect [10]. The maternal poliovirus antibodies have been shown to inhibit the immune response after priming vaccination with cIPVs in infants [2,3,4,5]. 

Limited studies of the sIPV that has widely been used in China since 2015 reported that a similar effect existed on dampening the immune response to sIPV vaccination [11,12,13,14]. However, there has been no data on the persistent inhibition effect of the maternal antibodies on the immunogenicity after the priming vaccination, and after the booster immunization with the fourth dose, let alone the quantification of the inhibitory effect of maternal poliovirus antibody, which usually requires a large sample size.

In this study, we aimed to analyze and quantify the instant and persistent effects of maternal poliovirus antibodies on the immune response to sIPV priming and booster vaccination, which helps to understand the inhibition effects of maternal poliovirus antibodies over time, and provides insights into optimizing IPV immunization strategies in the final stage of polio eradication.

## 2. Materials and Methods

### 2.1. Study Design and Participants

The randomized, double-blinded, parallel-controlled, phase IV clinical trial was conducted in Mile and Gejiu cities, Yunnan Province, China, from February 2018 to May 2020. The trial was approved by the Ethics Committee of the Yunnan Center for Disease Control and Prevention and was registered at ClinicalTrials.gov (NCT04224519). According to the IPV-only immunization schedule in the Expanded Program on Immunization (EPI) in China, participants received 3 doses of sIPVs at the ages of 2, 3, and 4 months as the priming immunization to explore the lot-to-lot consistency of commercial-scaled sIPVs. They were immunized with a 4th booster dose of the sIPV at the age of 18–24 months to analyze the immunogenicity of booster immunization.

Our previous studies indicated that two-sided 95% CIs for the GMT ratio among each lot for three poliovirus antibody types ranged from 0.80 to 1.39, falling within the equivalence range of 0.67–1.50 and indicating good immunogenicity consistency among the three commercial-scaled consecutive lots of sIPVs [14]. In this paper, we pooled the immunogenicity data of the three batch groups to further quantitatively analyze the instant and persistent effect of maternal antibodies on the immune response to sIPV priming, as well as the booster vaccination. 

### 2.2. Vaccination

sIPVs (IMBCAMS, Kunming) containing 30, 32, and 45 D-antigen units (DU) for types 1, 2, and 3, respectively, were packaged in vials (0.5 mL/vial) and were administered by intramuscular injection. Three consecutive commercial batches (Lots 20170931, 20170934, and 20171036) were used for the priming, and another commercial lot 201901007Q was administered for the booster vaccination in this trial. 

### 2.3. Immunogenicity Assessment

To assess the immunogenicity, blood samples of about 3 mL were collected on 0 day before and 30 days after the priming and booster vaccination. The endpoints for the immunogenicity assessment included the geometric mean titer (GMT). The microneutralization assays were performed by the National Institutes for Food and Drug Control (NIFDC) according to the method recommended by the WHO [15]. In brief, samples were serially diluted every two folds and neutralized for 3 h at 35 °C using a 100 cell culture infective dose 50% (CCID_50_) of Sabin strain poliovirus type 1, 2, or 3 in 96-well plates. HEp-2 cells were added to the serum/poliovirus mixture. After incubation for 7 days, cytopathic effects (CPEs) were observed. Poliovirus types 1, 2, and 3 specific neutralizing antibody titers were measured, and a titer of 1:8 before priming vaccination was considered to be positive [15], which indicates the presence of maternal antibodies. A titer of 1:8 was used for the categorization of maternal antibody negative (<1:8) and positive (≥1:8) groups.

### 2.4. Statistical Analysis

Statistical analyses were performed using SAS version 9.4 software (SAS Institute Inc., Cary, NC, USA). The antibody titers were calculated after logarithmic transformation (log 2) for the mean and its 95% confidence interval (CI) and then were calculated after antilog (2×) of the value for the GMTs and the 95% CIs. In order to explore the effect of maternal antibodies on the immune responses elicited by the sIPVs, the participants were categorized by two methods according to the antibody titers at baseline (before priming vaccination). The first method was to categorize participants into two groups: negative (<1:8) and positive (≥1:8), respectively, for the three poliovirus types; the second method was to categorize participants into four groups: “<1:8, 1:8 to 1:24, 1:32 to 1:192, ≥1:256” for type 1 and “<1:8, 1:8 to 1:24, 1:32 to 1:64, ≥1:96” for type 2 and type 3. Then, the neutralizing antibody titers after vaccination were compared among the two or four categorized groups using t-tests or one-way ANOVA tests after the log2 transformation of titers. Further comparisons between either of the four groups would be analyzed if the overall significance threshold of 0.05 was met. To quantify the effect of a maternal poliovirus antibody, the association between maternal antibody titers and post-vaccination antibody titers was estimated using linear regression models after the log2 transformation of antibody titers with the post-vaccination antibody titers as the dependent variable at each time point, respectively, and the maternal antibody titers as the independent variable. In the unadjusted model, no other covariates were adjusted in the linear regression model; in the adjusted model, the age and sex of the participants were adjusted as covariates. Thereafter, the antilog (2×) of the coefficients and the 95% CIs from the linear regression model were calculated as the geometric mean ratio (GMR), representing that every 2-fold increasing maternal poliovirus antibody titer may result in a (1-GMR) lower post-immunization antibody titer. All *p*-values were two-sided, and a value of <0.05 indicated statistical significance, except that the significance threshold was Bonferroni corrected to 0.008 (α’ = 0.05/6 = 0.008) in the further comparisons between any two of the four groups.

## 3. Results

### 3.1. Baseline of Participants in Groups with Different Maternal Poliovirus Antibody Titers in Prime and Booster Immunization

In this phase IV clinical trial, a total of 1200 participants were enrolled; the immunogenicity of 1140 participants after priming vaccination and the immune persistence of 1100 participants one year after priming, as well as the immunogenicity of 1100 participants after the booster vaccination, were assessed.

The maternal poliovirus antibody positive rates of the 1140 participants for poliovirus types 1, 2, and 3 were 61.8%, 47.9%, and 23.2%, respectively (Table 1), and 149 (13.1%) of the 1140 participants had positive maternal poliovirus antibodies for all the three types of polioviruses.

No significant difference in age or sex distribution was noticed between the two groups based on the first method, respectively, for poliovirus types 1, 2, and 3 of maternal antibodies (Table 1). There was also no significant difference in age or sex distribution among the four groups based on the second method, respectively, for poliovirus types 1 and 3 maternal antibodies (Table 2), except for a significant difference in sex distribution among the four groups (Table 2, *p* = 0.041) for poliovirus type 2. However, there was no significant difference in any two of the four groups after Bonferroni correction.

### 3.2. Comparisons of Antibody Titers after the Prime and Booster Vaccination in Groups with Different Maternal Poliovirus Antibody Titers

**Figure 1:**
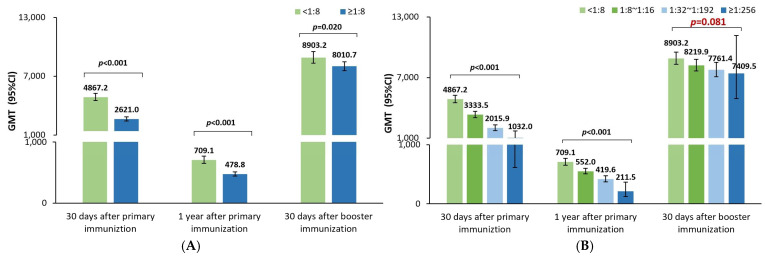
Comparison of the GMTs of neutralizing antibody against poliovirus type 1. (**A**) Comparisons of the neutralizing antibody titers between positive and negative maternal poliovirus antibody groups by using t-tests after the log2 transformation of the neutralizing antibody titers; the significance threshold was 0.05. (**B**) Comparisons of the neutralizing antibody titers among four groups with different maternal poliovirus antibody titers by using one-way ANOVA tests after the log2 transformation of the neutralizing antibody titers; the overall significance threshold among four groups was 0.05; if the overall significance met the significance threshold of 0.05, further comparisons between any two of the four groups were analyzed, as shown in Appendix A (Table A1).

**Figure 2:**
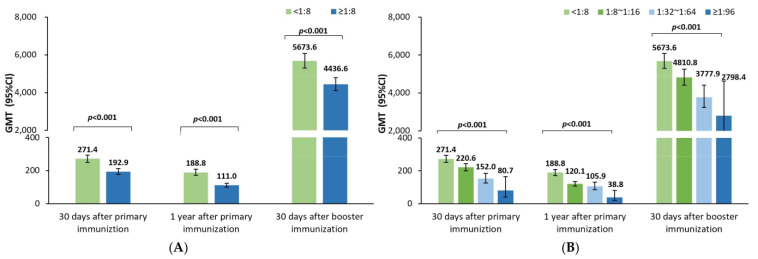
Comparison of the GMTs of neutralizing antibody against poliovirus type 2. (**A**) Comparisons of the neutralizing antibody titers between positive and negative maternal poliovirus antibody groups by using t-tests after the log2 transformation of the neutralizing antibody titers; the significance threshold was 0.05. (**B**) Comparisons of the neutralizing antibody titers among four groups with different maternal poliovirus antibody titers by using one-way ANOVA tests after the log2 transformation of the neutralizing antibody titers; the overall significance threshold among four groups was 0.05; if the overall significance met the significance threshold of 0.05, further comparisons between any two of the four groups were analyzed, as shown in Appendix A (Table A2).

**Figure 3:**
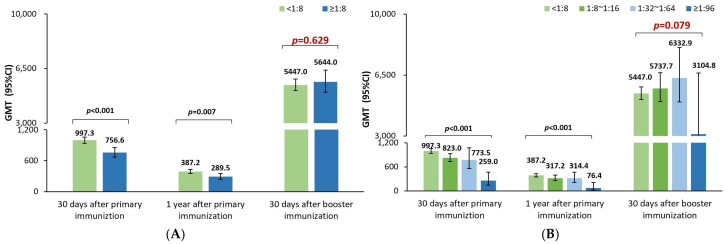
Comparison of the GMTs of neutralizing antibody against poliovirus type 3. (**A**) Comparisons of the neutralizing antibody titers between positive and negative maternal poliovirus antibody groups by using t-tests after the log2 transformation of the neutralizing antibody titers; the significance threshold was 0.05. (**B**) Comparisons of the neutralizing antibody titers among four groups with different maternal poliovirus antibody titers by using one-way ANOVA tests after the log2 transformation of the neutralizing antibody titers; the overall significance threshold among four groups was 0.05; if the overall significance met the significance threshold of 0.05, further comparisons between any two of the four groups were further analyzed, as shown in Appendix A (Table A3).

The GMTs at 30 days and one year after the priming vaccination were significantly lower in participants with positive rather than negative maternal poliovirus antibody groups for poliovirus types 1 (Figure 1A), 2 (Figure 2A), and 3 (Figure 3A). Moreover, the GMTs were gradually decreased in the four groups with the increasing maternal poliovirus antibody titers both at 30 days and one year after the priming vaccination for poliovirus type 1 (Figure 1B), type 2 (Figure 2B), and type 3 (Figure 3B) (all *p*-values < 0.001). 

However, at 30 days after the booster vaccination, no significant difference in GMTs was noticed between the positive and negative maternal poliovirus antibody groups for poliovirus type 3 (*p*-value = 0.629, Figure 3A) or among the four groups for poliovirus type 3 (*p*-value = 0.079, Figure 3B). Likely, no significant difference in GMTs was noticed among the four groups for poliovirus type 1 (*p*-value = 0.081, Figure 1B); but the GMTs were noticed to be significantly lower in the positive rather than negative maternal poliovirus antibody groups for poliovirus type 1 (*p*-value = 0.020, Figure 1A) at 30 days after the booster vaccination. Nevertheless, the same results were not shown for poliovirus type 2; there were still significantly lower GMTs in the positive rather than negative maternal poliovirus antibody groups (*p*-value < 0.001, Figure 2A), and the GMTs gradually decreased in the four groups at 30 days after the booster vaccination (*p*-value < 0.001, Figure 2B).

The antibody titers after the prime and booster vaccination by poliovirus-type specific maternal antibody titers are shown in Appendix A (Table A4, Table A5 and Table A6). Similar results were noticed by specific maternal poliovirus antibodies.

The above results most likely suggest that the early inhibition effect of maternal poliovirus antibodies on the immune response to sIPV priming vaccination could mostly be attenuated for type 1, partly offset for type 2, and completely attenuated for type 3 after the booster vaccination on children who were 18–24 months old.

### 3.3. Quantitative Analysis of the Maternal Antibody Inhibition Effect on the Immune Response Elicited by sIPV Prime and Booster Vaccination

At 30 days after the priming vaccination, the GMR was 0.79 (0.77–0.82), 0.85 (0.81–0.88), and 0.87(0.83–0.91), respectively, for poliovirus types 1, 2, and 3, indicating that every 2-fold increase in the maternal poliovirus antibody titer may result in 21%, 15%, and 13% lower postimmunization antibody titers against poliovirus types 1, 2, and 3 (Table 3, all *p*-values < 0.001).

One year after the priming vaccination, the GMR was 0.86 (0.83–0.89), 0.81 (0.77–0.85), and 0.86 (0.80–0.93), respectively, for poliovirus types 1, 2, and 3 (Table 3, all *p*-values < 0.001), indicating that the maternal poliovirus antibody continues to further affect the immune persistence of the sIPV priming immunization.

However, 30 days after the sIPV booster shot, no significant association was shown between the maternal antibody for poliovirus type 3 and the elicited antibody titers after the booster shot (Table 3, *p*-value = 0.348). Moreover, the GMR for poliovirus type 1 was changed from 0.79 (0.77–0.82) 30 days after the priming shot to 0.96 (0.94–0.99) after the booster shot, which was much closer to 1.0 (Table 3, *p*-value = 0.003), indicating that the negative effect of the maternal poliovirus antibody on the immune response was mostly weakened by the sIPV booster shot in children 18–24 months old. Interestingly, the GMR was 0.89 (0.86–0.93) (Table 3, *p*-value < 0.001) for type 2 with a little change from 0.85 after the priming shot to 0.89 after the booster shot, indicating that every 2-fold increase in the maternal poliovirus antibody titer may lead to an 11% lower antibody titer against poliovirus type 2, even after the sIPV booster shot, which likely suggests a partial offset of the inhibition effect of the maternal poliovirus antibody on the immune response by the sIPV booster shot.

## 4. Discussion

To our knowledge, this might be the first analysis of the persistent effect of the maternal poliovirus antibody titers on the immune response to the sIPV priming and booster vaccination in a large cohort of over 1000 infant participants that has been performed by quantitating the inhibition effects of maternal poliovirus antibodies on the immunogenicity after sIPV prime and booster vaccination. 

Like the previous studies investigating the effect of maternal poliovirus antibodies on the immune responses to the sIPV priming series in China [11,12,13,14], the poliovirus antibody GMTs 30 days after sIPV priming vaccination were noticed to be significantly lower in positive rather than negative maternal antibody participants in this study; and the GMTs also tended to gradually decrease with an increase in the maternal antibody titers for poliovirus types 1, 2, and 3 in this study, which was in line with the results of a post analysis of data from another sIPV phase I and phase II clinical trial in China [11]. Additionally, the GMRs were 0.79 (0.77–0.82), 0.85 (0.81–0.89), and 0.87 (0.83–0.91), respectively, for poliovirus types 1, 2, and 3, indicating that every 2-fold increase in the maternal poliovirus antibody titer may result in a 21%, 15%, and 13% lower postimmunization antibody titer against poliovirus types 1, 2, and 3 at 30 days after the sIPV priming vaccination. This finding was in alignment with the results in a meta-analysis after cIPV priming vaccination [10], which showed that the GMRs were 0.80 (0.78–0.83), 0.72 (0.69–0.74), and 0.78 (0.75–0.82), respectively, for poliovirus types 1, 2, and 3. Understanding the quantitative inhibition effect of the maternal poliovirus antibody on the sIPV priming vaccination is believed to help better optimize the IPV immunization strategy for eliciting and maintaining higher antibody levels against poliovirus, especially in later infancy.

As a consequence of the inhibition effect of the maternal poliovirus antibodies on the immune responses to the sIPV priming series, the GMTs were still significantly lower in the positive rather than the negative maternal poliovirus antibody participants one year after the sIPV priming vaccination. The GMRs were 0.86 (0.83–0.89), 0.81 (0.77–0.85), and 0.86 (0.80–0.93) for types 1, 2, and 3, representing that every 2-fold higher maternal poliovirus antibody titer may result in a 14%, 19%, and 13% lower antibody titer one year later. This was in partial alignment with the results from a meta-analysis reporting that the GMRs were 0.725 (0.684–0.768) and 0.692 (0.651–0.736) for types 1 and 2 and 0.939 (0.877–1.006) with no statistical significance for type 3 [10]. This difference in type 3 is presumably attributed to the sample size (unreported for the durability part) of the meta-analysis or the heterogeneity of different studies. However, it could still be concluded that the inhibition effect of the maternal poliovirus antibodies on the immune responses to IPV priming could further negatively influence the durability of poliovirus antibody titers for a period of 1 year.

Further, our study demonstrated that the poliovirus antibody GMTs were significantly lower for poliovirus types 1 and 2 after the sIPV booster shot in children 18–24 months old with positive rather than negative maternal antibodies, but surprisingly not for type 3. The GMRs were 0.96 (0.94–0.99), 0.89 (0.86–0.93), and 0.98 (0.93–1.03), respectively, for poliovirus types 1, 2, and 3, showing that every 2-fold higher maternal antibody titer may result in a 4%, 11%, and 0% lower antibody titers after the booster shot against poliovirus types 1, 2, and 3. Unlike the abundant studies of the maternal pertussis antibody on the immune response to the booster vaccination of pertussis vaccines [7,8], there were few studies of the inhibition of the maternal poliovirus antibodies on the immune response to the IPV booster vaccination, except for one meta-analysis of the 488 enrolled participants [10], showing GMRs of 0.90 (0.86–0.95), 0.82 (0.78–0.87), and 0.80 (0.75–0.84) for poliovirus types 1, 2, and 3, respectively. The difference between the three types of poliovirus characteristics, as well as their related antibody titers at baseline, may collectively contribute to eliciting different immune responses regarding the inhibition effects of maternal antibodies; however, the potential reasons need further exploration.

Interestingly, in our study, the inhibition effects of the maternal antibody titers in terms of the 1-GMRs were changed from 21%, 15%, and 13% to 14%, 19%, and 14% at 30 days and one year after the sIPV priming series, and further to 4%, 11%, and 0% at 30 days after the sIPV booster shot for poliovirus types 1, 2, and 3, which resulted from every 2-fold higher maternal poliovirus antibody titer. Obviously, a small difference in the inhibition effect was shown in the antibody titer decrease for poliovirus types 1, 2, and 3 at 30 days and one year after the sIPV priming series; however, after the booster shot, such persistent inhibition effects were almost reduced for poliovirus types 1 and 3 and partially reduced for type 2. The potential rationale for the prolonged persistent maternal antibody inhibition effect on poliovirus type 2 is presumably attributed to either the biological characteristics of Sabin strain 2 or the assumption of D antigen damage resulting from the formalin inactivation process [16,17] that leads to poorer elicited immunogenicity in terms of the lower antibody levels after the priming series compared with that of types 1 and 3. Thus, the inhibition effect of the maternal poliovirus antibody on the immunogenicity of the sIPV immunization was identified to exist until the day before the booster dose, i.e., 1 year after the prime series, and even continue to inhibit the immune response of poliovirus type 2 to the sIPV booster dose. 

Currently, in China, the licensed sIPV in an IPV-only schedule was 2-3-4 months for priming and 18 months for the booster to achieve rapid protection against the poliovirus in early age due to the current epidemiological settings with circulations of VDPV1 and VDPV3 from the routine EPI for poliomyelitis using the bivalent oral poliovirus vaccine (bOPV). There is also the threat of the importation of WPV from neighboring countries [18]. However, it is intended to implement an IPV-only schedule to prevent VDPV circulation at the final stage of polio eradication, as recommended by the WHO [19]. As OPV could elicit stronger nasopharyngeal mucosal immunity [20], particularly intestinal mucosal immunity, which could limit the poliovirus shedding from the intestine [21,22], higher antibody levels are required for IPV recipients to protect against poliovirus infection. Marine found that higher IPV-induced antibody levels (titers ≥ 1:128 for polio type 1) can reduce fecal excretion rates in a study involving families exposed to WPV1 [23]. It is reported that cVDPV2 infection and transmission were successfully stopped in response to a cVDPV2 outbreak in China during 2019–2021 due to the higher antibody levels elicited by the timely booster shot, which most likely suggests the potential effectiveness of the sIPV in cVDPV2 outbreaks [24]. Thus, maintaining higher poliovirus antibody levels in the IPV-only immunization program is of great importance for the final eradication of polio in the global world.

A review of enhanced potency IPV use in fifteen years concluded that antibody titers were often consistently higher in a 2-4-6-month schedule than that of the other three-dose schedules, i.e., a 2-3-4- or 3-4-5-month schedule [25]. Furthermore, previous studies have suggested that a wider spacing schedule of the second and third doses of vaccination may allow maternal antibody decay [10,26]. For example, the inhibition effects of maternal pertussis antibodies were attenuated in a 2-, 4-, and 6-month schedule as compared with a 2-, 3-, and 4-month schedule [10]. Taking into consideration these factors, it might be safe to conclude that a wider interval between sIPV doses might become an appropriate option in countries with no requirement for achieving rapid protection against poliovirus at the final stage of polio eradication. Further investigation of the wider spacing doses in priming and the booster is believed to be of great significance. 

## 5. Conclusions

This phase Ⅳ trial in a large cohort of children provided remarkable quantitative evidence of the persistent inhibition effects of the maternal poliovirus antibodies on the immune responses to poliovirus types 1, 2, and 3 from 30 days to 1 year after the sIPV priming series, and such inhibition effects were almost reduced for poliovirus types 1 and 3 and partially reduced for poliovirus type 2 at 30 days after the booster shot. A wider interval between the four sIPV doses might be a consideration for reducing the inhibition effects of the maternal antibodies and subsequently eliciting and maintaining higher antibody levels to protect against poliovirus transmission and infection at the final stage of polio eradication in the global world.

## Figures and Tables

**Table 1:** Baseline of participants in two groups with different maternal poliovirus antibody titers in prime and booster immunization.

Maternal Poliovirus Antibody Groups	Priming Immunization (*n* = 1140)				Booster Immunization (*n* = 1110)			
	Participant	Age, Month *	Sex, Male *	GMT	Participant	Age, Month *	Sex, Male *	GMT
Type 1								
Negative	436 (38.2%)	2.0 ± 0.0	214 (49.1%)	4.0	424 (38.5%)	18.3 ± 0.6	207 (48.8%)	4.0
Positive	704 (61.8%)	2.0 ± 0.0	334 (47.4%)	27.0	676 (61.5%)	18.3 ± 0.6	324 (47.9%)	26.8
Type 2								
Negative	594 (52.1%)	2.0 ± 0.0	281 (47.3%)	4.0	570 (51.8%)	18.3 ± 0.6	271 (47.5%)	4.0
Positive	546 (47.9%)	2.0 ± 0.0	267 (48.9%)	19.5	530 (48.2%)	18.3 ± 0.6	260 (49.1%)	19.5
Type 3								
Negative	876 (76.8%)	2.0 ± 0.0	411 (46.9%)	4.0	845 (76.8%)	18.3 ± 0.6	399 (47.2%)	4.0
Positive	264 (23.2%)	2.0 ± 0.0	137 (51.9%)	19.2	255 (23.2%)	18.3 ± 0.6	132 (51.8%)	19.3

Notes: * All *p*-values > 0.05.

**Table 2:** Baseline of participants in four groups with different maternal poliovirus antibody titers in prime and booster immunization.

Maternal Poliovirus Antibody Groups	Priming Immunization (*n* = 1140)				Booster Immunization (*n* = 1110)			
	Participant	Age, Month *	Sex, Male #	GMT	Participant	Age, Month *	Sex, Male *	GMT
Type 1								
Negative	436 (38.2%)	2.0 ± 0.0	214 (49.1%)	4.0	424 (38.5%)	18.3 ± 0.6	207 (48.8%)	4.0
[8, 24]	410 (36.0%)	2.0 ± 0.0	185 (45.1%)	13.8	395 (35.9%)	18.3 ± 0.6	179 (45.3%)	13.7
[32, 192]	262 (23.0%)	2.0 ± 0.0	128 (48.9%)	56.3	253 (23.0%)	18.3 ± 0.7	126 (49.8%)	56.5
≥256	32 (2.8%)	2.0 ± 0.0	21 (65.6%)	373.5	28 (2.6%)	18.2 ± 0.4	19 (67.9%)	384.5
Type 2								
Negative	594 (52.1%)	2.0 ± 0.0	281 (47.3%)	4.0	570 (51.8%)	18.3 ± 0.6	271 (47.5%)	4.0
[8, 24]	390 (34.2%)	2.0 ± 0.0	178 (45.6%)	13.0	381 (34.6%)	18.2 ± 0.5	175 (45.9%)	13.0
[32, 64]	132 (11.6%)	2.0 ± 0.0	72 (54.5%)	43.6	126 (11.5%)	18.4 ± 0.8	69 (54.8%)	44.0
≥96	24 (2.1%)	2.0 ± 0.0	17 (70.8%)	164.9	23 (2.1%)	18.4 ± 0.7	16 (69.6%)	176.2
Type 3								
Negative	876 (76.8%)	2.0 ± 0.0	411 (46.9%)	4.0	845 (76.8%)	18.3 ± 0.6	399 (47.2%)	4.0
[8, 24]	188 (16.5%)	2.0 ± 0.0	99 (52.7%)	11.8	182 (16.5%)	18.3 ± 0.6	96 (52.7%)	11.8
[32, 64]	60 (5.3%)	2.0 ± 0.0	28 (46.7%)	43.9	57 (5.2%)	18.4 ± 0.9	26 (45.6%)	43.8
≥96	16 (1.4%)	2.0 ± 0.0	10 (62.5%)	282.4	16 (1.5%)	18.0 ± 0.0	10 (62.5%)	282.4

Note: * All *p*-values > 0.05. # *p*-value > 0.05, except that the *p*-value was 0.041 for sex distribution among participants in four groups with different maternal poliovirus antibody statuses for type 2 in priming vaccination; however, there was no significant difference in any two of the four groups after Bonferroni correction (α’ = 0.05/6 = 0.008).

**Table 3:** Association of the maternal poliovirus antibodies and the poliovirus type-specific neutralizing antibody titers after priming and booster vaccination (FAS).

Poliovirus Neutralizing Antibody	Unadjusted Model			Adjusted Model	
	GMR (95% CI)	*p*-Value *		GMR (95% CI)	*p*-Value *
30 days after priming vaccination with 3 doses of the sIPV					
Type 1	0.79 (0.77–0.82)	<0.001		0.79 (0.77–0.82) ^a^	<0.001
Type 2	0.85 (0.81–0.88)	<0.001		0.85 (0.81–0.89) ^a^	<0.001
Type 3	0.87 (0.83–0.91)	<0.001		0.87 (0.83–0.91) ^a^	<0.001
1 year after priming vaccination the 3 doses of the sIPV					
Type 1	0.86 (0.83–0.88)	<0.001		0.86 (0.83–0.89) ^b^	<0.001
Type 2	0.80 (0.76–0.85)	<0.001		0.81 (0.77–0.85) ^b^	<0.001
Type 3	0.86 (0.80–0.93)	<0.001		0.86 (0.80–0.93) ^b^	<0.001
30 days after booster vaccination with the 4th dose of the sIPV					
Type 1	0.96 (0.94–0.99)	0.002		0.96 (0.94–0.99) ^b^	0.003
Type 2	0.90 (0.86–0.93)	<0.001		0.89 (0.86–0.93) ^b^	<0.001
Type 3	0.97 (0.93–1.03)	0.338		0.98 (0.93–1.03) ^b^	0.348

Note: The unadjusted model was not adjusted for any other covariates; the adjusted model, ^a^, was adjusted for the age of receiving the first dose and the sex of the participants; ^b^, adjusted for the age of receiving the booster dose and the sex of the participants. * The significance threshold was 0.05.

## Data Availability

The data presented in the current study are available from the corresponding author upon reasonable request.

## Appendix A

**Table A1:** *p*-values of comparisons on neutralizing antibody titers between any two of the four categorized groups against poliovirus type 1.

Maternal Antibody Groups of Poliovirus Type 1	30 Days after Priming Vaccination				1 Year after Priming Vaccination				30 Days after Booster Vaccination			
	Negative	[8, 24]	[32, 192]	≥256	Negative	[8, 24]	[32, 192]	≥256	Negative	[8, 24]	[32, 192]	≥256
Negative	-	<0.001	<0.001	<0.001	-	<0.001	<0.001	<0.001	NA	NA	NA	NA
[8, 24]		-	<0.001	<0.001		-	0.001	<0.001		NA	NA	NA
[32, 192]			-	0.002			-	0.001			NA	NA
≥256				-				-				NA

Note: The significance threshold was 0.008 between any two of the four groups after Bonferroni correction (α’ = 0.05/6 = 0.008). NA, not applicable.

**Table A2:** *p*-values of comparisons on neutralizing antibody titers between any two of the four categorized groups against poliovirus type 2.

Maternal Antibody Groups of Poliovirus Type 2	30 Days after Priming Vaccination				1 Year after Priming Vaccination				30 Days after Booster Vaccination			
	Negative	[8, 24]	[32, 64]	≥96	Negative	[8, 24]	[32, 64]	≥96	Negative	[8, 24]	[32, 64]	≥96
Negative	-	0.012	<0.001	<0.001	-	<0.001	<0.001	<0.001	-	0.024	<0.001	0.001
[8, 24]		-	0.002	<0.001		-	1.000	<0.001		-	0.039	0.021
[32, 64]			-	0.032			-	0.001			-	0.751
≥96				-				-				-

Note: The significance threshold was 0.008 between any two of the four groups after Bonferroni correction (α’ = 0.05/6 = 0.008).

**Table A3:** *p*-values of comparisons on neutralizing antibody titers between any two of the four categorized groups against poliovirus type 3.

Maternal Antibody Groups of Poliovirus Type 3	30 Days after Priming Vaccination				1 Year after Priming Vaccination				30 Days after Booster Vaccination			
	Negative	[8, 24]	[32, 64]	≥96	Negative	[8, 24]	[32, 64]	≥96	Negative	[8, 24]	[32, 64]	≥96
Negative	-	0.068	0.260	<0.001	-	0.621	1.000	<0.001	NA	NA	NA	NA
[8, 24]		-	1.000	<0.001		-	1.000	0.002		NA	NA	NA
[32, 64]			-	<0.001			-	0.005			NA	NA
≥96				-				-				NA

Note: The significance threshold was 0.008 between any two of the four groups after Bonferroni correction (α’ = 0.05/6 = 0.008). NA, not applicable.

**Table A4:** Poliovirus antibody titers after prime and booster vaccination by specific maternal antibody titers for poliovirus type 1.

Maternal Antibody Titer	30 Days after Prime Vaccination			1 Year after Prime Vaccination			30 Days after Booster Vaccination	
	*n*	Antibody Titers		*n*	Antibody Titers		*n*	Antibody Titers
<1:8	436	4096 (256, 32,768)		424	768 (32, 8192)		424	8192 (1536, 49,152)
1:8	113	4096 (384, 32,768)		111	768 (128, 3072)		111	8192 (1536, 49,152)
1:12	87	4096 (512, 32,768)		83	768 (32, 6144)		83	8192 (768, 32,768)
1:16	107	3072 (256, 16,384)		101	384 (48, 6144)		101	8192 (1536, 32,768)
1:24	103	3072 (256, 32,768)		100	512 (48, 3072)		100	8192 (1024, 24,576)
1:32	70	2048 (384, 32,768)		67	384 (48, 3072)		67	8192 (1536, 24,576)
1:48	79	2048 (64, 32,768)		77	384 (24, 6144)		77	8192 (1536, 49,152)
1:64	60	2048 (192, 16,384)		58	384 (24, 3072)		58	8192 (1536, 24,576)
1:96	20	2560 (48, 16,384)		18	384 (96, 1536)		18	12,288 (1536, 24,576)
1:128	16	2048 (64, 8192)		16	384 (12, 3072)		16	6144 (1536, 32,768)
1:192	17	2048 (128, 6144)		17	384 (12, 2048)		17	8192 (1536, 24,576)
1:256	14	1792 (48, 6144)		11	384 (48, 1536)		11	12,288 (1536, 24,576)
1:384	8	1408 (64, 12288)		8	384 (2, 1536)		8	10,240 (384, 24,576)
1:512	7	768 (192, 6144)		6	384 (48, 768)		6	7168 (1536, 32,768)
1:768	2	448 (128, 768)		2	96 (96, 96)		2	11,264 (6144, 16,384)
1:1024	0	NA		0	NA		0	NA
1:1536	1	6144 (6144, 6144)		1	384 (384, 384)		1	4096 (4096, 4096)

Note: Data are median (Min, Max) for antibody titers. NA, not applicable.

**Table A5:** Poliovirus antibody titers after prime and booster vaccination by specific maternal antibody titers for poliovirus type 2.

Maternal Antibody Titer	30 Days after Prime Vaccination			1 Year after Prime Vaccination			30 Days after Booster Vaccination	
	*n*	Antibody Titers		*n*	Antibody Titers		*n*	Antibody Titers
<1:8	594	256 (4, 8192) †		570	192 (2, 3072)		570	6144 (96, 24,576)
1:8	112	256 (24, 8192)		109	192 (6, 1536)		109	6144 (768, 24,576)
1:12	120	224 (12, 3072)		117	96 (2, 2048)		117	6144 (384, 24,576)
1:16	79	256 (16, 2048)		79	96 (12, 512)		79	4096 (384, 24,576)
1:24	79	192 (24, 2048)		76	128 (6, 1536)		76	4096 (384, 24,576)
1:32	57	192 (12, 2048)		52	96 (6, 1536)		52	6144 (192, 24,576)
1:48	39	128 (8, 1024)		39	96 (2, 1536)		39	3072 (1024, 8192)
1:64	36	192 (12, 1536)		35	96 (2, 768)		35	3072 (384, 16,384)
1:96	11	384 (12, 1536)		9	96 (6, 1024)		9	3072 (384, 24,576)
1:128	3	12 (8, 12)		3	24 (2, 48)		3	2048 (384, 2048)
1:192	3	64 (64, 768)		3	48 (48, 192)		3	4096 (2048, 6144)
1:256	3	192 (4, 256) §		4	30 (2, 384)		4	3584 (192, 6144)
1:384	0	NA		0	NA		0	NA
1:512	3	64 (64, 96)		3	32 (2, 128)		3	6144 (768, 8192)
1:768	1	8 (8, 8)		1	12 (12, 12)		1	6144 (6144, 6144)
1:1024	0	NA		0	NA		0	NA
1:1536	0	NA		0	NA		0	NA

Note: Data are median (Min, Max) for antibody titers. NA, not applicable. † One participant’s antibody titer of poliovirus type 2 remained 1:4 before and after vaccination. § One participant’s antibody titer of poliovirus type 2 decreased from 1:256 before vaccination to 1:4 after priming vaccination.

**Table A6:** Poliovirus antibody titers after prime and booster vaccination by specific maternal antibody titers for poliovirus type 3.

Maternal Antibody Titer	30 Days after Prime Vaccination			1 Year after Prime Vaccination			30 Days after Booster Vaccination	
	*n*	Antibody Titers		*n*	Antibody Titers		*n*	Antibody Titers
<1:8	876	1024 (16, 32,768)		845	512 (2, 8192)		845	6144 (96, 49,152)
1:8	81	768 (192, 8192)		78	384 (8, 6144)		78	6144 (48, 49,152)
1:12	46	768 (128, 3072)		45	384 (8, 8192)		45	6144 (1536, 49,152)
1:16	33	768 (128, 4096)		32	320 (2, 6144)		32	6144 (1536, 32,768)
1:24	28	512 (64, 6144)		27	256 (12, 4096)		27	3072 (768, 12,288)
1:32	23	768 (48, 6144)		22	384 (6, 3072)		22	6144 (384, 49,152)
1:48	23	1024 (96, 8192)		22	448 (48, 4096)		22	6144 (1536, 32,768)
1:64	14	512 (12, 4096)		13	128 (12, 1536)		13	4096 (1536, 12,288)
1:96	3	384 (96, 1024)		3	192 (48, 384)		3	3072 (768, 6144)
1:128	2	204 (24, 384)		2	130 (4, 256)		2	4864 (1536, 8192)
1:192	4	304 (48, 1024)		4	112 (6, 512)		4	3840 (384, 24,576)
1:256	2	640 (512, 768)		2	520 (16, 1024)		2	6336 (384, 12,288)
1:384	0	NA		0	NA		0	NA
1:512	1	256 (256, 256)		1	768 (768, 768)		1	4096 (4096, 4096)
1:768	0	NA		0	NA		0	NA
1:1024	2	432 (96, 768)		2	50 (4, 96)		2	3264 (384, 6144)
1:1536	2	256 (256, 256)		2	136 (16, 256)		2	17,920 (3072, 32,768)

Note: Data are median (Min, Max) for antibody titers. NA, not applicable.
